# Characterization and whole genome sequencing of closely related multidrug-resistant *Salmonella enterica* serovar Heidelberg isolates from imported poultry meat in the Netherlands

**DOI:** 10.1371/journal.pone.0219795

**Published:** 2019-07-22

**Authors:** Redmar R. van den Berg, Serge Dissel, Michel L. B. A. Rapallini, Coen C. van der Weijden, Ben Wit, Raymond Heymans

**Affiliations:** Netherlands Food and Consumer Product Safety Authority, Consumer and Safety Division, Laboratory Food and Feed Safety, WB Wageningen, the Netherlands; Cornell University, UNITED STATES

## Abstract

Multidrug-resistant *Salmonella enterica* serovar Heidelberg isolates are frequently recovered in the Netherlands from poultry meat imported from South America. Our aim was to retrospectively assess the characteristics of the antimicrobial determinants, gene content and the clonal relatedness of 122 unique *S*. Heidelberg isolates from chicken meat from Brazil (n = 119) and Argentina (n = 3) that were imported between 2010 and 2015. These isolates were subjected to antimicrobial susceptibility testing, PCR and Illumina HiSeq2500 whole genome sequencing. Draft genomes were assembled to assess the gene content, and the phylogenetic relationships between isolates were determined using single nucleotide polymorphisms. Ciprofloxacin-resistance was identified in 98.4% of the isolates and 83.7% isolates showed resistance to the extended-spectrum cephalosporins cefotaxime and ceftazidime (83.6% and 82.8% respectively). Of the latter, 97.1% exhibited an AmpC phenotype and contained *bla*_CMY-2_, whereas the remaining three isolates contained an extended spectrum beta-lactamase. Of the 99 extended-spectrum cephalosporins-resistant isolates harboring CMY-2 plasmids, 56.6% contained the incompatibility group I1 replicon. Phylogenetic cluster analysis showed that all isolates from Brazil clustered together, with 49% occurring in clusters larger than 5 isolates that revealed intra-cluster similarities based on geographical location and/or resistance profiles. The remaining isolates were classified in smaller clusters or as singletons, highlighting the large diversity of *S*. Heidelberg in the poultry chain in Brazil that was revealed by this study. Considering the potential public health risk associated with multidrug-resistant *S*. Heidelberg in imported poultry, collaborative whole genome sequencing-based surveillance is needed to monitor the spread, pathogenic properties and epidemiological distribution of these isolates.

## Introduction

*Salmonella enterica* serovar Heidelberg is highly prevalent in North America and a major cause of foodborne salmonellosis in humans [1]. In the USA, *S*. Heidelberg has been identified as one of the predominant serotypes in poultry, with several clones circulating in multistate outbreaks that resulted from the consumption of contaminated poultry products [2, 3]. Less is known about the prevalence of *S*. Heidelberg in poultry in South America. Yet, several studies reported the detection of multidrug-resistant (MDR) *S*. Heidelberg in poultry in a number of Latin American countries [4–13]. Although *S*. Heidelberg has been reported less frequently in Europe than in North America [14], the recent report of extended-spectrum cephalosporin (ESC) resistant *S*. Heidelberg strains in poultry meat in the Netherlands is reason for concern [15].

*S*. Heidelberg has high morbidity rates and is typically characterized by a self-limiting gastro-enteritis [16, 17]. In vulnerable patients, such as young children, the elderly and immunocompromised patients, this can occasionally develop into an invasive infection that requires antibiotic treatment [2]. Since ESCs are commonly used for the treatment of such life threatening infections, ESC resistant strains pose a potential public health risk. Therefore, efficient monitoring and surveillance using a robust, fast and highly discriminatory genotyping technique is essential for early identification of these foodborne pathogens and to facilitate the implementation of effective prevention and intervention strategies. Whole genome sequencing (WGS) provides the means for such extensive surveillance and source tracing within various populations and settings. Nowadays, WGS is increasingly applied as a molecular epidemiological tool for the identification and surveillance of foodborne pathogens and to assist in outbreak investigation [18–20]. WGS provides very high discriminating power to differentiate clinical and epidemiological relevant strains, and allows for detailed molecular characterization. With the advent of cost-effective, fast and reliable WGS technologies, it is now possible to perform simultaneous identification, characterization, evaluation and genotyping of bacterial isolates using a single genome sequence [21].

The Netherlands Food and Consumer Product Safety Authority monitors the import of poultry from third countries as part of the requirements under European Union council directive 97/78/EC. In 2010, the first multidrug-resistant (MDR) *S*. Heidelberg isolates emerged from raw poultry meat preparations imported from South America, mainly from Brazil, the main exporter of chicken to the Netherlands. Disturbingly, a substantial number of these isolates were resistant to extended-spectrum cephalosporin (ESC) [22]. Therefore, a retrospective study was conducted to assess the clonal relatedness of these imported MDR *S*. Heidelberg isolates using WGS-based single nucleotide polymorphism (SNP) analysis. To develop a reliable SNP genotyping method and assess its performance, a set of carefully selected control samples and read data from a number of publically available *S*. Heidelberg genomes from several geographical locations, from a similar timeframe, were included. De novo assembly of *S*. Heidelberg genomes was performed to identify plasmid replicons and to examine the correlation between known antimicrobial resistance determinants and antimicrobial resistance phenotypes.

## Materials and methods

### Bacterial isolates and control samples

The dataset in this study consisted of 134 multidrug-resistant *S*. *Heidelberg* isolates from imported raw chicken meat preparations from different locations in Brazil (n = 131) and Argentina (n = 3) that were collected from 2010 to 2015 (Table 1).

**Table 1:** Overview of the different *S*. Heidelberg isolates, unique isolates and SNP profiles from chicken meat imported from Brazil and Argentina included in this study.

	Country	Isolates (n)	Unique isolates (n)	SNP profiles in RAxML tree (n)
	Brazil	114	114	114
	Argentina	2	2	2
**Controls**				
Batch A	Brazil	5	1 (isolate was randomly selected)	5
Batch B	Brazil	5	1 (isolate was randomly selected)	5
Batch C	Brazil	5	1 (isolate was randomly selected)	5
Duplicate A	Brazil	1	1	2 (the isolate was sequenced twice)
Duplicate B	Argentina	1	1	2 (the isolate was sequenced twice)
Triplicate	Brazil	1	1	3 (the isolate was sequenced three times)
	**Total**	**134**	**122**	**138**
				19 (SNP profiles from public databases)
				2 (outgroup *S*. Senftenberg)
				1 (reference NC_011083)
			**Total**	**160**

A set of control samples was included to act as internal controls (Table 1). First, controls were included which consisted of isolates from three distinct shipments of poultry, referred to as “batches”, which arrived as a single shipment on the same date from the same processing plant. Two of these batches were sampled eight days apart in 2014 and one batch was sampled in 2015. Five bacterial isolates were recovered from each individual batch, making a total of 15 isolates. To prevent calculation bias, as the isolates in each separate batch were expected to be near identical *S*. Heidelberg variants, only one randomly selected isolate from each batch was included in the counts and statistics mentioned throughout the study. The second part of the control samples consisted of individual isolates that were sequenced multiple times. One *S*. Heidelberg isolate from Brazil (2013) was taken from frozen stock and sequenced twice (indicated as Duplicate A). Next, one *S*. Heidelberg isolate from Argentina (2015) was subcultured and a single colony was selected. The genome sequence of the same DNA extract was determined twice to identify potential WGS artefacts (indicated as Duplicate B). Finally, one *S*. Heidelberg isolate from Brazil (2012) was selected to be included in each of the three sequencing runs performed for this study (indicated as Triplicate).

In this study, we considered 114 isolates from Brazil and two isolates from Argentina. Additionally, one isolate from each batch (A, B and C; n = 3) was added, as well as the three control isolates that were sequenced multiple times (Duplicate A, Duplicate B and the Triplicate). In total, we analysed 122 unique isolates that were recovered from imported poultry. Any statistics reported throughout this paper will refer only to these 122 unique isolates, unless explicitly stated otherwise.

In total, 134 *S*. Heidelberg isolates from Brazil and Argentina (including the batches; n = 15) were analysed by Whole Genome Sequencing, generating 138 SNP profiles (including batch A, B and C, Duplicate A and B, and the Triplicate) that were included in a phylogenetic tree. All isolates included in this study were sequenced to a mean read depth of at least 41. To assess the performance of the WGS-based SNP analysis the read data of 19 *S*. Heidelberg isolates from various publicly available sources (see S1 Table in the supplemental material) were included. From the study of Bekal et al. 2016, the genome data (ebi.ac.uk 100K Pathogen Genome Project) of 5 *S*. Heidelberg isolates from each of the three different Canadian outbreaks, which occurred in 2012, 2013 and 2014, were selected [23]. Thus, from each outbreak five isolates were randomly selected and included in this study. In addition, the genome data of two *S*. Heidelberg isolates from Colombia [7] collected from fecal content and chicken meat (2013, 2012, respectively) and two isolates from Thailand (source and collection date unknown) were included in this study (ebi.ac.uk 100K Pathogen Genome Project). Two *Salmonella enterica* serovar Senftenberg isolates were included in the phylogenetic analysis to serve as an outgroup.

### Bacterial culturing, antimicrobial susceptibility testing, and plasmid analysis

Cryotubes containing *S*. Heidelberg isolates were taken from the -80°C freezer for subculturing. One bead from each cryotube was transferred on tryptic soy agar (TSA) and incubated overnight for 22h at 37°C. Next, a single colony was subcultured to TSA and incubated overnight for 22h at 37°C. Of each bacterial isolate a 0.5 McFarland suspension was prepared containing 5 ml sterilized water. Next, 55 μl suspension was added to 11 ml Brain-Heart Infusion Broth (BHI; Biotrading, Mijdrecht, The Netherlands) and incubated overnight for 18h at 37°C. Antimicrobial susceptibility testing was performed with the Sensititre Vizion system (Thermo Scientific, MA, USA) using the EUMVS2 (2010 to 2013) and EUVSEC (2014 and on) panels according to the ISO 20776 2006 recommendations. Cefotaxime and/or ceftazidime resistant samples (cefotaxime MIC of >0.5 mg/L, ceftazidime MIC of >2 mg/L) were further examined with the confirmatory EUVSEC2 panel for extended spectrum β-lactamases (ESBLs). In this study, we examined a selection of the following 11 antimicrobial agents that were part of both the EUMVS2 and EUVSEC panels: cefotaxime (CTX), ceftazidime (CAZ), ciprofloxacin (CIP), ampicillin (AMP), chloramphenicol (CHL), colistin (CST), gentamicin (GEN), nalidixic acid (NAL), sulfamethoxazole (SMX), tetracycline (TET) and trimethoprim (TMP). The antimicrobial resistance breakpoints were determined according to the recommendations of the European Committee on Antimicrobial Susceptibility Testing (EUCAST, 2017).

A subset of 45 ESC-resistant *S*. Heidelberg isolates, that presented the AmpC and ESBL phenotypes, was selected for plasmid transformation and PCR-based replicon typing as described by Liakopoulos et al. 2016 [15].

### DNA library preparation, genome sequencing, and assembly

The complete DNA content of each bacterial isolate was extracted from overnight cultures using the DNeasy Blood & Tissue Kit (Qiagen, Hilden, Germany) and sequenced using the HiSeq 2500 platform (Illumina, San Diego, USA) according to the manufacturer’s protocol.

SNP profiles were generated by mapping the Illumina reads against *S*. Heidelberg reference genome SL476 (NC_011083, excluding plasmids) based on the Genome Analysis ToolKit (GATK) best practises. In short, Illumina adapters and low quality reads were removed using Trimmomatic (v0.35). The resulting reads were mapped to *S*. Heidelberg reference genome SL476 using BWA-mem (v0.7.12–5) and PCR duplicates were removed via Picard Tools (v1.113–2). Next, indel realignment was performed, genomic VCF files were generated using the GATK (v3.60) in ERC mode, and SNP variants were called using GATK GenotypeGVCFs. SNPs with a low coverage (read depth <10) and conflicting reads (<90% of reads agree with the called genotype) were not included. The filtered SNPs were then concatenated into SNP profiles.

To determine the gene content, trimmed reads were assembled using ABySS (v1.9.01), and BLAST (v2.2.31) was used to extract genes of interest from the assembly. Blast hits with a nucleotide identity below 80% were rejected. The MLST scheme from pubmlst.org was used to predict the sequence types. The downloaded databases of ResFinder (2018-09-10) and PlasmidFinder (2018-11-06), available at https://cge.cbs.dtu.dk/services/, were used with in-house developed tools to determine the antibiotic resistance determinants and plasmid types, respectively. PointFinder (commit bd50f0b) and the PointFinder database (commit d1413d2) were used to identify point mutations in chromosomal genes that confer antibiotic resistance [24].

### Phylogenetic cluster analysis

Randomized Axelerated Maximum Likelihood (RAxML v8.2.4.1) was used for maximum likelihood phylogenetic tree estimation [25]. In short, the Gamma model for rate heterogeneity was used to allow for varying rates of evolution in different regions of the profile. The Lewis ascertainment bias correction was used to compensate for the fact that the SNP profile only includes variable positions. The bootstrap convergence of the phylogenetic tree was calculated and the extended majority rule consensus was applied to determine the required number of bootstrap iterations [26].

## Results

### Antimicrobial resistance

Antimicrobial susceptibility testing of the 122 unique *S*. Heidelberg isolates showed that all isolates from Brazil (n = 119) and two of the three isolates from Argentina were multidrug-resistant to four or more classes of antimicrobial agents (Table 2). Ciprofloxacin resistance (MIC of >0.064 mg/L) was identified in 98.4% (120 of 122) of the isolates and extended spectrum cephalosporin (ESC) resistance was identified in the majority of the isolates; 83.6% (102/122) were resistant to cefotaxime (MIC of >0.5 mg/L) and 82.8% (101/122) showed resistance to ceftazidime (MIC of >2 mg/L). Of the cefotaxime-resistant isolates, 99.0% (101/102) also showed resistance to ceftazidime.

**Table 2:** Characterization of the antibiotic resistance phenotypes of 122 *S*. Heidelberg isolates from imported poultry meat collected between 2010 and 2015.

Antimicrobial resistance phenotypes^a^											*bla*_gene_	Resistance genes on plasmids	Country	No. of resistant isolates (%)
CTX	CAZ	CIP	AMP	NAL	SMX	TET					CMY-2	*fosA*, *sul2*, *tetA*	Brazil	92 (75.4)
												*fosA*, *QnrB19*, *sul2*, *tetA*		3 (2.5)
CTX	CAZ	CIP	AMP	NAL	SMX	TET			GEN		CMY-2	*fosA*, *aadA1*, *aac(3)-Via*, *sul2*, *tetA*	Brazil	1 (0.8)
CTX	CAZ	CIP	AMP	NAL	SMX	TET		CST			CTX-M-8	*fosA*, *sul2*, *tetA*	Brazil	1 (0.8)
CTX	CAZ	CIP	AMP	NAL	SMX	TET	CHL			TMP	CMY-2	*fosA*, *aadA8*, *cmlA1*, *sul1*, *sul3*, *tetA*, *dfrA15*	Brazil	1 (0.8)
CTX	CAZ	CIP	AMP	NAL							CMY-2	*fosA*, *QnrB19*	Argentina	1 (0.8)
CTX	CAZ		AMP		SMX	TET					CTX-M-2	*fosA*, *sul1*, *tetA*	Brazil	1 (0.8)
CTX	CAZ		AMP								CMY-2	*fosA*	Argentina	1 (0.8)
CTX		CIP	AMP	NAL	SMX	TET			GEN		CTX-M-8, TEM-1B	*fosA*, *aadA1*, *strA*, *strB*, *aac(3)-Via*, *mph(B)*, *sul1*, *sul2*, *tetA*	Brazil	1 (0.8)
		CIP	AMP	NAL		TET	CHL				TEM-1B	*fosA*, *catA1*, *QnrB19*, *tetA*	Argentina	1 (0.8)
		CIP		NAL	SMX	TET			GEN		-	*fosA*, *aadA1*, *aac(3)-Via*, *sul1*, *sul2*, *tetA*	Brazil	5 (4.1)
												*fosA*, *aadA1*, *aac(3)-Via*, *sul1*, *sul2*, *tetA*, *aph(3’)Ia*		1(0.8)
		CIP		NAL	SMX	TET					-	*fosA*, *sul2*, *tetA*	Brazil	13 (10.7)
													**Total (n)**	122 (100)

^a^ CTX: Cefotaxime; CAZ: Ceftazidime; CIP: Ciprofloxacin; AMP: Ampicillin; NAL: Nalidixic Acid; SMX: Sulfamethoxazole; TET: Tetracycline; CHL: Chloramphenicol; CST: Colistin; GEN: Gentamycin; TMP: Trimethoprim

Percentages of resistance against the other antimicrobial agents tested: AMP (84.4%; 103/122), NAL (98.4%; 120/122), SMX (97,5%; 119/122), TET (98.4%; 120/122), CHL (1.6%; 2/122), CST (0.8%; 1/122), GEN (6.6%; 7/122) and TMP (0.8%; 1/122)

Assessment of the antimicrobial resistance phenotypes of these isolates showed that 97.1% (99 of 102) ESC-resistant isolates exhibited an AmpC phenotype. Genome analysis revealed the presence of *bla*_CMY-2_ in all AmpC *S*. Heidelberg isolates and no discrepant results were identified following PCR confirmation. Of the three remaining ESC-resistant isolates that contained no *bla*_CMY-2_, one isolate harbored *bla*_CTX-M-2_ while another isolate harbored *bla*_CTX-M-8_. In the third isolate, association of *bla*_CTX-M-8_ and *bla*_TEM-1_ genes was revealed. In 20 of the 122 isolates that were susceptible to cefotaxime and ceftazidime, no *β*-lactamases were identified with the exception of one Argentinian isolate that harbored *bla*_TEM-1_, which confers resistance to several penicillins. This isolate was the only isolate of these 20 isolates with resistance to ampicillin. Overall, 99 of 122 (81.1%) of the multidrug-resistant *S*. Heidelberg isolates from Brazil and Argentina harbored *bla*_CMY-2_ and exhibited an AmpC *β*-lactamase phenotype.

### Identification of antimicrobial resistance genes and plasmids

A comparison between the phenotypic antimicrobial resistance data and the resistance gene WGS data showed that in addition to the *bla* genes, 16 different plasmid-associated antibiotic resistance genes were identified (Table 2). Of the antibiotics included in this study, the identified resistance genes were expected to produce resistance to sulfamethoxazole, tetracycline, chloramphenicol, gentamycin, ciprofloxacin and trimethoprim. Our results confirmed that all resistance phenotypes correlated with the genotypes identified for these antibiotic agents. Ciprofloxacin resistance is often induced by mutations in *gyrA* and *parC* and in *Salmonella* these mutations are also related to nalidixic acid resistance. All of the 122 *S*. Heidelberg isolates had a chromosomal *parC* mutation (T57S). Of these, 118 (96.7%) isolates carried a chromosomal *gyrA* mutation (S83F) as well. Interestingly, three of the 118 (2.5%) isolates had also a plasmid that carried *QnrB19*. Two (1.6%) ciprofloxacin-resistant isolated lacked the *gyrA* mutation but harbored the *QnrB19* gene. In the two (1.6%) ciprofloxacin-susceptible isolates, both *QnrB19* and *gyrA* mutations were absent. *QnrB19* is known to confer a low level of resistance to ciprofloxacin while also facilitating mutations in *gyrA* [27]. In the colistin-resistant isolate no *mcr* genes (1 to 5) were identified and the MIC of >4 mg/L is just one step above the cut-off (MIC of >2 mg/L).

After *de novo* assembly of the sequencing data, the presence of several plasmid replicons was revealed by using the PlasmidFinder database (Table 3). Co-carriage of plasmids was common in this isolate collection. Large multidrug-resistant plasmids of the IncX1 and IncA/C incompatibility groups were most frequently identified and were present in 120 of 122 (98.4%) and 118 of 122 (96.7%) of the isolates, respectively. IncI1 was present in 65 of 122 (53.3%) of the isolates. Of the 99 ESC-resistant isolates harboring *bla*_CMY-2_, IncI1 was identified in 56 (56.6%) isolates. To verify whether the plasmid replicons of these incompatibility groups identified were present, transformation experiments were conducted on an arbitrarily selected subset of 45 isolates. All identified IncI1 replicons (n = 32) were confirmed and no discrepant results were identified. Ninety-seven (98.0%) of these 99 isolates carried IncX1 and IncA/C plasmids of which 54/97 (54.5%) co-carried IncI1. Notably, the four different plasmid profiles that occur in 91.0% of the isolates (111/122) exhibited 11 different antimicrobial phenotypes. Additional genomic analysis of the 65 isolates containing an IncI1 plasmid replicon revealed that the primary plasmid multilocus sequence type (pMLST) was ST12 (n = 56; 86.2%). The remaining isolates exhibited ST178 (n = 4; 6.2%), ST113 (n = 3; 4.6%), ST26 (n = 1; 1.5%) and one of the isolates from Argentina showed a combination of known plasmid alleles that was not part of any of the known types, suggesting this plasmid is a novel sequence type.

**Table 3:** Plasmid profiles among 122 *S*. Heidelberg isolates used in this study.

Plasmids		Antimicrobial resistance phenotypes (n)	*bla*_gene_	IncI1 pMLST (ST)	No. of isolates (%)
Incompatibility groups	Small plasmids				
IncX1, IncA/C, IncI1	ColpVC	CTX, CAZ, CIP, AMP, NAL, SMX, TET (43)	CMY-2	ST12	43 (35.2)
		CTX, CAZ, CIP, AMP, NAL, SMX, TET, CHL, TMP (1)	CMY-2	ST12	1 (0.8)
		CTX, CAZ, CIP, AMP, NAL, SMX, TET, CST (1)	CTX-M-8	ST113	1 (0.8)
		CIP, NAL, SMX, TET, GEN (4)	-	ST178	4 (3.3)
		CIP, NAL, SMX, TET (1)	-	ST113	1 (0.8)
IncX1, IncA/C, IncI1	ColpVC, ColRNAI	CTX, CAZ, CIP, AMP, NAL, SMX, TET (7)	CMY-2	ST12	7 (5.7)
		CIP, NAL, SMX, TET, GEN (1)	-	ST12	1 (0.8)
IncX1, IncA/C, IncI1	ColpVC, Col156	CTX, CAZ, CIP, AMP, NAL, SMX, TET, GEN (1)	CMY-2	ST26	1 (0.8)
IncX1, IncA/C, IncI1, IncFII	ColpVC	CTX, CAZ, CIP, AMP, NAL, SMX, TET (1)	CMY-2	ST12	1 (0.8)
IncX1, IncA/C, IncI1, IncX4	ColpVC	CTX, CAZ, CIP, AMP, NAL, SMX, TET (1)	CMY-2	ST12	1 (0.8)
IncX1, IncA/C	ColpVC	CTX, CAZ, CIP, AMP, NAL, SMX, TET (28)	CMY-2	-	28 (23.0)
		CIP, NAL, SMX, TET (11)	-	-	11 (9.0)
IncX1, IncA/C	ColpVC, Col(BS512)	CTX, CAZ, CIP, AMP, NAL, SMX, TET (1)	CMY-2	-	1 (0.8)
IncX1, IncA/C	ColpVC, ColRNAI	CTX, CAZ, CIP, AMP, NAL, SMX, TET (13)	CMY-2	-	13 (10.7)
		CIP, NAL, SMX, TET (1)	-	-	1 (0.8)
IncX1, IncA/C, IncFII	ColpVC, ColRNAI	CTX, CAZ, CIP, AMP, NAL, SMX, TET (1)	CMY-2	-	1 (0.8)
IncX1, IncA/C, IncHI2	ColpVC	CIP, NAL, SMX, TET, GEN (1)	-	-	1 (0.8)
IncX1, IncA/C, IncI1, IncHI2, IncQ	ColRNAI	CTX, CIP, AMP, NAL, SMX, TET, GEN (1)	CTX-M-8, TEM-1B	ST113	1 (0.8)
IncX1, IncI1	-	CIP, AMP, NAL, TET, CHL (1)	TEM-1B	unknown type	1 (0.8)
IncX1, IncHI2	-	CTX, CAZ, AMP, SMX, TET (1)	CTX-M-2	-	1 (0.8)
IncI1	ColpVC, Col156	CTX, CAZ, CIP, AMP, NAL (1)	CMY-2	ST12	1 (0.8)
IncI1	Col156	CTX, CAZ, AMP (1)	CMY-2	ST12	1 (0.8)

### MLST analysis

Genome data analysis to establish the multilocus sequence type (MLST) revealed that all but one of the isolates from Brazil and Argentina were indistinguishable, as all of these isolates were assigned sequence type 15 (n = 121). The only exception was a single isolate from Brazil that was more distantly related to the other Brazilian isolates, and was assigned ST4632 (n = 1) due to a single SNP in *thrA1*. This outlier was confirmed by resequencing, and all other control samples were assigned MLST type 15 (Table 4).

**Table 4:** Characteristics of the resistance determinants and the distance between the SNP profile of each control sample included in this study.

Control samples	Isolate No.	Country	State/province	Resistance phenotype	*bla*_gene_	De novo Plasmids	MLST (ST)	Max.SNP distance
Duplicate A	I	Brazil	Santa Catarina	CTX, CAZ, AMP, SMX, TET	CTX-M-2	IncX1, IncHI2	4632	0
	II	Brazil	Santa Catarina	CTX, CAZ, AMP, SMX, TET	CTX-M-2	IncX1, IncHI2	4632	
Duplicate B	I	Argentina	Entre Ríos	CTX, CAZ, AMP	CMY-2	IncI1, Col156	15	0
	II	Argentina	Entre Ríos	CTX, CAZ, AMP	CMY-2	IncI1, Col156	15	
Triplicate	I	Brazil	Paraná	CTX, CAZ, CIP, AMP, NAL, SMX, TET	CMY-2	IncX1, IncA/C, IncI1, ColpVC, ColRNAI	15	1
	II	Brazil	Paraná	CTX, CAZ, CIP, AMP, NAL, SMX, TET	CMY-2	IncX1, IncA/C, IncI1, ColpVC, ColRNAI	15	
	III	Brazil	Paraná	CTX, CAZ, CIP, AMP, NAL, SMX, TET	CMY-2	IncX1, IncA/C, IncI1, ColpVC, ColRNAI	15	
Batch A	I	Brazil	Santa Catarina	CTX, CAZ, CIP, AMP, NAL, SMX, TET	CMY-2	IncX1, IncA/C, ColpVC	15	1
	II	Brazil	Santa Catarina	CTX, CAZ, CIP, AMP, NAL, SMX, TET	CMY-2	IncX1, IncA/C, ColpVC	15	
	III	Brazil	Santa Catarina	CTX, CAZ, CIP, AMP, NAL, SMX, TET	CMY-2	IncX1, IncA/C, ColpVC	15	
	IV	Brazil	Santa Catarina	CTX, CAZ, CIP, AMP, NAL, SMX, TET	CMY-2	IncX1, IncA/C, ColpVC	15	
	V	Brazil	Santa Catarina	CTX, CAZ, CIP, AMP, NAL, SMX, TET	CMY-2	IncX1, IncA/C, ColpVC	15	
Batch B	I	Brazil	Santa Catarina	CTX, CAZ, CIP, AMP, NAL, SMX, TET	CMY-2	IncX1, IncA/C, ColpVC	15	2
	II	Brazil	Santa Catarina	CTX, CAZ, CIP, AMP, NAL, SMX, TET	CMY-2	IncX1, IncA/C, ColpVC	15	
	III	Brazil	Santa Catarina	CTX, CAZ, CIP, AMP, NAL, SMX, TET	CMY-2	IncX1, IncA/C, ColpVC	15	
	IV	Brazil	Santa Catarina	CTX, CAZ, CIP, AMP, NAL, SMX, TET	CMY-2	IncX1, IncA/C, ColpVC	15	
	V	Brazil	Santa Catarina	CTX, CAZ, CIP, AMP, NAL, SMX, TET	CMY-2	IncX1, IncA/C, ColpVC	15	
Batch C	I	Brazil	Santa Catarina	CIP, NAL, SMX, TET	-	IncX1, IncA/C, ColpVC, IncI1	15	2
	II	Brazil	Santa Catarina	CIP, NAL, SMX, TET	-	IncX1, IncA/C, ColpVC, IncI1	15	
	III	Brazil	Santa Catarina	CIP, NAL, SMX, TET	-	IncX1, IncA/C, ColpVC, IncI1	15	
	IV	Brazil	Santa Catarina	CIP, NAL, SMX, TET	-	IncX1, IncA/C, ColpVC, IncI1	15	
	V	Brazil	Santa Catarina	CIP, NAL, SMX, TET	-	IncX1, IncA/C, ColpVC, IncI1	15	

### Cluster analysis of the control set

**Fig 1:**
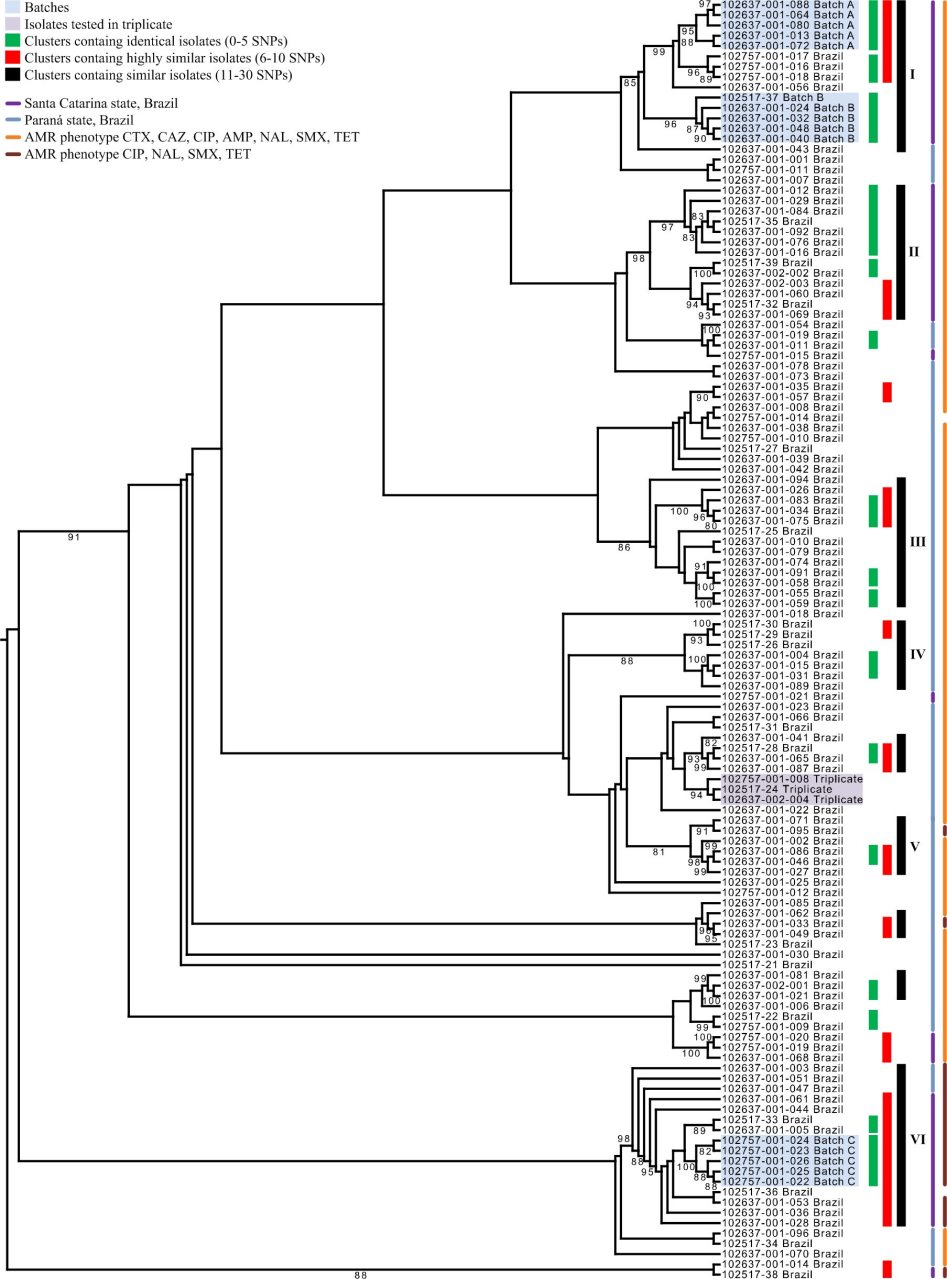
The upper half of the RAxML tree. This illustration contains 124 (110 unique isolates from Brazil) of the 138 *S*. Heidelberg SNP profiles (122 unique isolates) included in this study. Cluster analysis was performed to display the genetic relationship between the SNP profiles of the *S*. Heidelberg variants. Only clusters with bootstrap support values ≥80 were indicated. The SNP profiles and clusters in the RAxML tree are color coded according to the legend.

To assess the validity of the high resolution cluster classification based on SNP analysis, we first investigated the clustering of the control samples. The control samples, derived from the sampling of three distinct shipments of poultry meat, assigned batch A, B, and C (Table 4), were grouped together with a bootstrap support values of 95, 96, and 100 and with a maximal SNP distance between isolates of 1, 2, and 2 SNPs, respectively (Fig 1).

**Fig 2:**
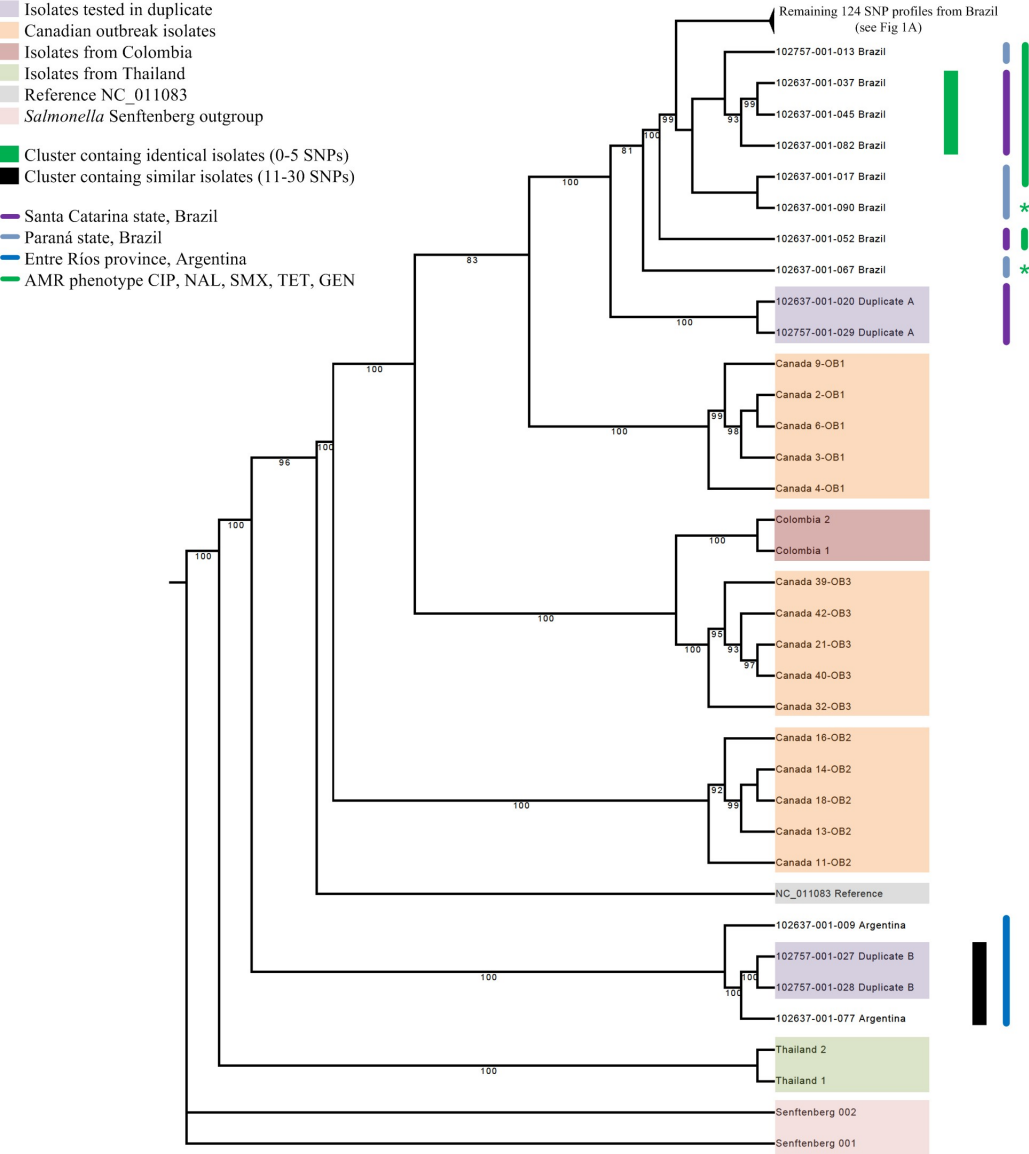
The lower half of the RAxML tree. **This illustration contains 14 (12 unique isolates from Brazil and Argentina) of the 138 *S*. Heidelberg SNP profiles (122 unique isolates) included in this study.** Cluster analysis was performed to display the genetic relationship between the *S*. Heidelberg variants from Brazil and Argentina, as well as the 19 SNP profiles from publicly available genomes (Canada, Colombia and Thailand). Only clusters with bootstrap support values ≥80 were indicated. *S*. Senftenberg was included as an outgroup. The SNP profiles and clusters in the RAxML tree are color coded according to the legend. In isolates 102637-001-067 and 102637-001-090 (illustrated by an green asterisk *) gentamycin resistance was also identified but the resistance profiles differed from Antimicrobial resistance phenotype CIP, NAL, SMX, TET, GEN as these two isolates showed additional resistance to AMP, CTX and AMP, CTX, CAZ, respectively.

Next, the classification of the control samples that were sequenced multiple times (Table 4) was assessed as illustrated in Figs 1 and 2.

The SNP profiles of the control sample duplicate A from Brazil, which was sequenced twice, clustered together with a bootstrap support value of 100, and 0 SNPs. The SNP profiles from duplicate B, derived from the same DNA extraction step, showed zero SNPs difference and were paired together with a bootstrap support value of 100. The three SNP profiles that were derived from the single isolate indicated as triplicate, were placed together with a bootstrap support value of 94, and a maximal distance of one SNP. We therefore decided to set the cut-off value for identical isolates at 5 SNPs, since we are not able to reliably differentiate between isolates that differ less than five SNPs, using our pipeline. Next we set out to establish a terminology to describe clusters of more distantly related isolates. Note that this terminology was only used when the reliability of a cluster had been established, specifically if the bootstrap support value was at least 80. In order of increasing distance, we described isolates as identical (0–5 SNPs), highly similar (6–10 SNPs) or similar (11–30 SNPs).

The arbitrary aspect of these cut-off values mean that they are well suited for the analysis of this sample set using our pipeline and parameters. Therefore, these cut-off values should not be taken as a general guideline, but rather as a situational convention [28].

### Cluster analysis

The RAxML tree in S1 Fig shows that all *S*. Heidelberg isolates formed a clade, with an average SNP distance of 40,076 SNPs from the out-group that comprised *Salmonella enterica* subsp. *enterica* serovar Senftenberg. An average SNP distance of 27 and 29 SNPs was found between the isolates from Brazil and between the isolates from Argentina, respectively, whereas 131 SNPs were identified between the isolates of both countries. An average number of 7350, 100, 34, 85, 69 SNPs were found between the Brazilian *S*. Heidelberg isolates and isolates from Thailand, Colombia and Canadian outbreak I, II and III, respectively.

Fig 2 also shows that our method is able to differentiate between the SNP profiles from isolates from various geographical areas and outbreaks by grouping them with good bootstrap support. All SNP profiles from Brazil cluster exclusively together, as do the SNP profiles from Argentina, Colombia, Thailand and the three Canadian outbreaks.

Eighty-four of the 122 isolates (68.9%) were assigned to at least one cluster, while the remaining 38 isolates (31.1%) were singletons. Note that isolates can be a member of multiple clusters, e.g. when a highly similar cluster contains two or more identical isolates. The isolates from Brazil and Argentina were classified in a total of 14 clusters (not counting the batches) with identical isolates (0 to 5 SNPs between isolates), ranging in size from 2 to 7 isolates. The period between sampling in these clusters varied from the same day to isolates that were sampled 20 months apart. A total of 38 (31.1%) isolates were assigned to 11 clusters with highly similar isolates (a SNP difference between 6 and 10), ranging from 2 to 9 isolates. The period between sampling in these clusters varied from the same day to isolates that were sampled approximately 23 months apart. When we considered similar clusters, with a SNP difference of 11 to 30 SNPs between isolates, a total of 66 (54.1%) isolates were assigned to ten clusters with similar isolates, ranging from 2 to 13 isolates. The period between sampling in these clusters varied from approximately three months to almost 5.5 years between the isolates from Argentina. In the following section we only describe six clusters (I to VI) that contain at least 5 similar isolates (11–30 SNPs, see Table 5).

**Table 5:** Characteristics of 58 unique isolates that were classified in six substantial clusters (n≥5 isolates) with similar isolates (11–30 SNPs).

Clusters						
	I	II	III	IV	V	VI
No. isolates^a^	7^b^	13	13	7	6	12^c^
State/province (n)	Santa Catarina (6)	Santa Catarina (13)	Paraná (13)	Paraná (7)	Santa Catarina (1)	Santa Catarina (9)
	Paraná (1)				Paraná (5)	Paraná (3)
Maximal SNP distance	25	16	26	20	28	14
Location poultry processing plant (n)	Chapecó (3)	Chapecó (13)	Toledo (13)	Toledo (7)	Concórdia (1)	Chapecó (9)
	Concórdia (3)				Toledo (5)	Toledo (3)
	Toledo (1)					
Approx. sampling period in months	13	9	6	2.5	3.5	27.5
Years (n)	2013 (1)	2013 (8)	2012 (1)	2013 (7)	2013 (6)	2013 (9)
	2014 (6)	2014 (5)	2013 (12)			2014 (2)
						2015 (1)
antimicrobial resistance phenotypes (n)	CTX, CAZ, CIP, AMP, NAL, SMX, TET (7)	CTX, CAZ, CIP, AMP, NAL, SMX, TET (13)	CTX, CAZ, CIP, AMP, NAL, SMX, TET (13)	CTX, CAZ, CIP, AMP, NAL, SMX, TET (7)	CTX, CAZ, CIP, AMP, NAL, SMX, TET (6)	CIP, NAL, SMX, TET (11)
						CTX,CAZ, CIP, AMP, NAL, SMX, TET, CST (1)^d^
Plasmid replicons (n)	IncX, IncA/C, ColpVC (6)	IncX, IncA/C, ColpVC (6)	IncX, IncA/C, ColpVC (1)	IncX, IncA/C, ColpVC, IncI1 (6)	IncX, IncA/C, ColpVC, IncI1 (2)	IncX, IncA/C, ColpVC (9)
	IncX, IncA/C, ColpVC, ColRNAI (1)	IncX, IncA/C, ColpVC, IncI1 (7)^e^	IncX, IncA/C, ColpVC, IncI1 (5)	IncX, IncA/C, ColpVC, ColRNAI (1)	IncX, IncA/C, ColpVC, ColRNAI (2)	IncX, IncA/C, ColpVC, IncI1 (2)
			IncX, IncA/C, ColpVC, ColRNAI (4)		IncX, IncA/C, ColpVC, IncI1, ColRNAI (1)	IncX, IncA/C, ColpVC, ColRNAI (1)
			IncX, IncA/C, ColpVC, IncI1, ColRNAI (2)		IncX, IncA/C, ColpVC, IncI1, IncFII (1)	
			IncX, IncA/C, ColpVC, IncI1, IncX4 (1)			

^a^ Isolates tested in duplicate or triplicate and isolates in batches were counted as one isolate to prevent calculation bias.

^b^ including two batches

^c^ including one batch

^d^ *bla*_CTX-M-8_

^e^ isolates with plasmid profile IncX, IncA/C, ColpVC, IncI1 all belong to a cluster with identical isolates which is a subcluster of cluster II

The 119 unique isolates from chicken meat from Brazil originated from two adjacent states, Paraná (n = 79) and Santa Catarina (n = 40). The isolates from chicken meat that originated from Paraná came from six different poultry processing plants, located in Cafelândia, Cascavel, Marechal Cândido Rondon, Palotina, Rolândia and Toledo. The isolates from chicken meat that originated from Santa Catarina came from three different poultry processing plants, located in Chapecó, Concórdia and Nova Veneza.

In the clusters with similar isolates, clusters I, III, IV, V and VI, a total of 1/7 (14.3%), 13/13 (100%), 7/7 (100%), 5/6 (83.3%) and 3/12 (25.0%) of the isolates originated from Paraná, respectively. These clusters all contained isolates from chicken meat that originated from a poultry processing plant in the city of Toledo in the Paraná state. The remaining isolates from Paraná were classified in two small clusters with identical isolates, four clusters with highly similar isolates, and as 34 (43.0%) singletons. These clusters and singletons were unique and thus not part of a larger cluster.

We found that the isolates from chicken meat that originated from Santa Catarina (n = 40) showed a strong tendency to group together (Figs 1 and 2). In the clusters with similar isolates, clusters I, II, V and VI, a total of 6/7 (85.7%), 13/13 (100%), 1/6 (16.7%) and 9/12 (75.0%) of the isolates from chicken meat originated from Santa Catarina, respectively. Interestingly, the subcluster with highly similar isolates that was part of cluster VI, exclusively contained isolates from Santa Catarina. Cluster I contained three isolates from chicken meat from Chapecó and three isolates from Concórdia that all originated from the Santa Catarina state. Cluster II exclusively contained isolates from a poultry processing plant in Chapecó. Cluster V only contained one isolate from Concórdia and cluster VI contained nine unique isolates from two poultry processing plants in the city of Chapecó. The remaining isolates from chicken meat that originated from Santa Catarina (n = 11) were classified in one cluster with identical isolates (n = 3), two additional clusters with highly similar isolates (n = 4), and as four singletons. The three isolates from chicken meat from Argentina originated from the city of Concepción del Uruguay in the Entre Ríos province.

The three primary antimicrobial resistance phenotypes (Table 2) also grouped together in the tree as illustrated in Figs 1 and 2. Clusters I to V shared the most common ESC resistant antimicrobial resistance phenotype AMP, CTX, CAZ, CIP, NAL, SMX, TET. All the isolates in cluster VI contained the phenotype CIP, NAL, SMX, TET with the exception of one ESC resistant isolate that exhibited an AmpC *β*-lactamase phenotype, harbored *bla*_CTX-M-8_ and displayed the AMP, CTX, CAZ, CIP, CST, NAL, SMX, TET phenotype. None of the other isolates contained *bla*_CMY-2_ or were ESC resistant. The phenotype CIP, NAL, SMX, TET was only identified in two other isolates. One isolate belonged to a cluster with three similar isolates and one isolates was classified in a highly similar cluster of only two isolates. Isolates with antimicrobial resistance phenotype CIP, GEN, NAL, SMX, TET clearly grouped together, even though only three of the six isolates were classified to a small cluster with identical isolates. Six different plasmid profiles were identified in clusters I to VI, ranging from two to five per cluster. Notably, seven of the 13 isolates in cluster II, belong to a subcluster with identical isolates that contained plasmid replicons IncX, IncA/C, ColpVC and IncI1. The sampling period between the isolates in clusters I to VI varied from approximately 2.5 to 27.5 months. The three Argentinian *S*. Heidelberg isolates grouped together despite a delay of almost 5 years and 7 months between the first and last isolate sampled. A maximal SNP distance of 63 SNPs between the isolates indicates they are more distantly related.

The Canadian outbreak isolates were classified in three separate clusters. The maximal SNP distance between the isolates of each separate outbreak cluster was one SNP. The isolates from Thailand and Colombia were grouped in pairs with a maximal SNP distance of 7 and 43 between isolates, respectively. Interestingly, the Colombian isolates were grouped next to the isolates of Canadian outbreak III and a maximal SNP distance of 75 SNPs was found between the Colombian and the Canadian outbreak III isolates. This makes these clusters distantly related according to the terminology used for the phylogenetic classification.

## Discussion

In this retrospective study, we show the emergence of clonally related multidrug-resistant (MDR) *S*. Heidelberg isolates from poultry meat that was imported to the Netherlands between 2010 and 2015. Most of these isolates originated from ten different cities of two adjacent states from the Southern part of Brazil, Paraná (n = 79) and Santa Catarina (n = 40) and were all but one assigned ST15, a sequence type commonly identified among *S*. Heidelberg strains and often reported in Asia, Europe and the Americas in both food and human isolates [5, 6, 29]. Interestingly, the only exception was the most distant isolate relative to the other isolates from Brazil that was assigned ST4632 based on a single SNP in one of the MLST genes. Nearly all isolates exhibited fluoroquinolone resistance (98.4%), a worrying observation as these antibiotics are considered the first-line antimicrobial agents for the treatment of salmonellosis [30–32]. In 2016, the EFSA also reported a substantial number (64.7%) of ciprofloxacin-resistant isolates (breakpoint MIC of >0.064 μg/ml, EUCAST) from broiler meat in 16 of the 19 European Member States that participated. In contrast, the NARMS reported that in 2015 only 0.7% of the retail chicken meat samples in the United States were resistant to ciprofloxacin (breakpoint MIC of ≥0.12 μg/ml, CLSI) [33, 34]. Even more disturbingly, 83.6% of the isolates were resistant to cefotaxime. Since ESCs are often administered in severe cases of salmonellosis in risk groups, or in cases of infection caused by fluoroquinolone-resistant strains, these results show the serious limitations concerning effective treatment in case of severe infection with these *S*. Heidelberg strains imported from Brazil [35]. In the isolates imported from Brazil, 81.5% displayed an AmpC phenotype and *bla*_CMY-2_ was the most frequently identified *β*-lactamase in these isolates. Although limited information is available on the prevalence of *bla*_CMY-2_ in *Salmonella* in Brazil, Giuriatti et al. 2017 reported CMY-2*-*positive *S*. Heidelberg isolates that exhibited ESC resistance from Paraná state, Brazil, which is where most isolates in this study were imported from [10]. Moreover, *bla*_CMY-2_ was reported to be widely distributed in *Escherichia coli* isolates detected in Brazilian chicken carcasses [36]. Besides *bla*_CMY-2_, several ESBL genes (e.g. TEM-, SHV- and CTX-M-type ESBLs) have been associated with the appearance of ESC resistance in *Salmonella* [37]. In our study, we identified only four isolates containing ESBL genes (*bla*_CTX-M-2,_ *bla*_TEM-1,_ and *bla*_CTX-M-8_) using genome analysis. Despite the limited number of ESBLs detected in this study, the CTX-M-2 and CTX-M-8 enzymes are nowadays highly prevalent in South America and contribute to ESC resistance in several *Salmonella enterica* serovars [8, 38, 39]. Until recently CTX-M-8 enzymes were rarely identified in *S*. Heidelberg, but a recent report also described the presence of *bla*_CTX-M-8_ in an *S*. Heidelberg isolate from Brazilian poultry meat [40]. Notably, no ESC resistance was identified in absence of a corresponding *bla*_CMY-2_ or ESBL gene in this study.

It appears these resistance determinants are widely disseminated among various *Salmonella* serovars in Brazil, including *S*. Heidelberg [8, 38, 40, 41]. The identification of these clonally related multidrug-resistant *S*. Heidelberg variants originating from several Brazilian states suggests the successful expansion and persistence of these apparently biologically fit variants. The extensive use of antibiotics in the poultry industry for the treatment and prevention of disease, as well as growth promotion in broilers, can create favorable conditions for the acquisition of resistance determinants [42–44]. Commensal microbiota in the gastrointestinal tract serve as an especially dangerous reservoir for antimicrobial resistance as these bacteria can rapidly be altered by antibiotic pressure [45]. Interspecies horizontal transfer and/or inter-serovar exchange of resistance determinants can provide *Salmonella* with a selective advantage to facilitate spread and persistence. Although the logistics concerning the Brazilian poultry industry are not entirely clear, contamination with MDR *S*. Heidelberg could occur during (inter)national exchange of broiler stock and/or equipment between poultry farms or during the transport and storage of poultry carcasses [9]. The environmental persistence of MDR *S*. Heidelberg in poultry farms may allow for continued exposure and the often asymptomatic infection of live poultry [46, 47]. Another reason that allowed these *S*. Heidelberg variants to survive might be inadequate hygienic procedures at the poultry processing plants. Especially problems with the pre-chilling procedure, which has been reported as a critical processing step to eradicate *Salmonella* spp., can contribute to the contamination of the final poultry products with viable *Salmonella spp*. [48]. In turn, this might also lead to the persistence of pathogens on processing equipment, a known risk for cross contamination [48, 49].

Of the plasmids belonging to known incompatibility groups, transmissible plasmids IncX1, IncA/C and IncI1 were most common. These plasmids are often associated with persistence, as well as carrying virulence and antimicrobial resistance in poultry by disseminating ESBL genes [50, 51]. Our results also strongly suggest that most of the *bla*_CMY-2_ identified were located on the plasmids of the incompatibility groups IncI1 and IncA/C, although in some cases a chromosomal association may exist, as transformation experiments and replicon typing were only performed on a representative subset of samples collected [29, 52–54].

To further assess the potential variation between these clonally related isolates, we performed high-resolution subtyping by using 138 SNP profiles (including the control samples) for phylogenetic cluster analysis. The correct classification of the control samples was a strong indication that the phylogenetic allocation of the filtered SNP profiles provided the means to discriminate between different *S*. Heidelberg variants while maintaining meaningful groups.

For the purpose of discussion, we adopted an arbitrary terminology for cluster classification, partially based on the differences of the SNP profiles between matching control samples, to assess three different levels of genotyping resolution. Interestingly, instead of large groupings of Brazilian *S*. Heidelberg isolates, many small (sub)clusters with less than five isolates were observed. Only when we considered clusters which contained isolates that differed between 11 to 30 SNPs, six substantial clusters (of at least five isolates) with good bootstrap support were identified, meaning that only 58 isolates were assigned to a substantial cluster. Thus, even after using the latter cluster classification, 31.1% (37/119) of the Brazilian *S*. Heidelberg isolates appeared as a singleton or were assigned to a small cluster (20.2%; 24/119). The limited number of substantial clusters strongly suggests that in a large clonally related population of *S*. Heidelberg strains, subtyping resulted in the identification of numerous *S*. Heidelberg variants that circulate in the states of Paraná and Santa Catarina. It appears that in Brazil several *S*. Heidelberg lineages, presumable of common ancestry, contained an epidemic strain that underwent clonal expansion and diversification. Resistance determinants might have been acquired by ancestral strains, while maintaining biological fitness, conferring antimicrobial resistance to the epidemic strains. Microevolution during the expansion of the epidemic clones could explain the high number of *S*. Heidelberg variants identified [55]. Moreover, it is likely that the substantial number of variants we presented in this study are an underestimation of the real variation of *S*. Heidelberg isolates that are circulating in the Brazilian poultry chain, taking into account the high number of singletons and the fact that a relatively small sample size was presented provided by random sampling of imports from the southern part of Brazil.

We were able to differentiate six clusters, containing five or more isolates, which showed similarities in geographical location and antimicrobial resistance phenotype. Assessment of the geographical location of the isolates showed that the isolates from different poultry processing plants in Santa Catarina had a tendency to cluster together, which is clearly illustrated by the separate clusters I, II and VI. These results suggest that, although not exclusively, a persistent *S*. Heidelberg clone was circulating on the poultry production farms and/or poultry processing plants located in this state. Although the resistance markers might not be ultimately conclusive, the classification of cluster VI was reinforced by the antimicrobial resistance phenotypic data. Eleven of the 12 (91.7%) isolates in cluster VI were susceptible for the ESCs tested and showed an MDR phenotype (CIP, NAL, SMX, TET) that diverged from the dominant MDR phenotype found in this study (CTX, CAZ, CIP, AMP, NAL, SMX, TET). The isolates in this cluster clearly contained an *S*. Heidelberg variant that is, with a maximal SNP distance of 54 SNPs, more distantly related to the isolates in clusters I to V. However, we are aware that the lack of profound epidemiological data concerning our isolate collection hindered in-depth epidemiological characterization of these high-resolution (sub)clusters.

The *S*. Heidelberg isolates from Argentina, Thailand, Colombia and Canada showed a strong geographical signature and could be clearly differentiated from the Brazilian isolates, despite the fact that almost all Brazilian isolates were classified as ST15. Each of the three individual Argentinian isolates presented a unique and divergent MDR phenotype compared to the Brazilian MDR phenotypes, as well as an average SNP distance of 90 SNPs, indicating that the Argentinian *S*. Heidelberg isolates had no direct link to the Brazilian isolates. The isolates from different Canadian outbreaks were assigned to three different clusters, which is consistent with the findings of Bekal et al. 2016, supporting the validity of the SNP analysis in this study [23]. Notably, the Colombian isolates that were collected from poultry fecal matter and chicken meat, appear to be closest related (an average of 65 SNPs) to the human clinical isolates from Canadian outbreak III. The link between the consumption of poultry products and human infection by *Salmonella* is well documented. Several studies reported that the presence of *Salmonella* in healthy poultry is considered as one of the major transmission risks for human infection by direct transmission or via eggs and poultry meat [2, 56, 57].

In this study, we demonstrated the high discriminative power of WGS-based SNP analysis to distinguish clonally related *S*. Heidelberg isolates from imported chicken meat that were multidrug-resistant against medically important antibiotics, including ESC’s. We revealed a high genetic diversity among the *S*. Heidelberg variants that circulate in Brazil, while maintaining meaningful groups of *S*. Heidelberg variants that were outbreak related and originated from other geographical locations over a substantial period of time. The phylogenetic relationships suggest that there is evidence that implies the variants from Brazil originated from a common ancestor. Yet, the different plasmid profiles also suggest that the ESC resistance can be attributed to the wide dissemination of several plasmids that are transferred by horizontal transmission between identified and still unidentified *S*. Heidelberg variants as well as interspecies plasmid transfer. Although we are confident that our method is applicable for the efficient monitoring of local outbreaks and the tracing of variants in a contaminated production environment, we are aware that the current data set is limited in size. Studies with a larger sample size and extensive epidemiological data, collected over a longer time-span, are required to determine suitability of the method for large scale analysis. Nevertheless, our data provides additional insight into the spread of MDR *S*. Heidelberg variants and the reservoir of resistance determinants in the Brazilian poultry industry. These findings are serious reason for concern and should be addressed. The MDR character of these *S*. Heidelberg variants pose a potential health hazard, and a collaborative WGS-based surveillance by food safety authorities is needed to provide a deeper understanding of the pathogenicity, antimicrobial resistance and epidemiological spread of these isolates.

## Supporting information

S1 Fig Complete RAxML tree of 138 *S*. Heidelberg SNP profiles (122 unique isolates) generated by whole genome sequencing. (TIF)

S1 Table Public read data that was included in this study. The first column is the sample name used in this study, the remaining columns are data taken from the SRA archive.
(XLSX)

## References

[1] Hoffmann M, Zhao S, Pettengill J, Luo Y, Monday SR, Abbott J, et al Comparative genomic analysis and virulence differences in closely related salmonella enterica serotype heidelberg isolates from humans, retail meats, and animals. Genome Biol Evol. 2014;6(5): 1046–68. 10.1093/gbe/evu079 24732280 PMC4040988

[2] Antunes P, Mourao J, Campos J, Peixe L. Salmonellosis: the role of poultry meat. Clin Microbiol Infect. 2016;22(2): 110–21. 10.1016/j.cmi.2015.12.004 26708671

[3] Gieraltowski L, Higa J, Peralta V, Green A, Schwensohn C, Rosen H, et al National Outbreak of Multidrug Resistant Salmonella Heidelberg Infections Linked to a Single Poultry Company. PloS one. 2016;11(9): e0162369 10.1371/journal.pone.0162369 27631492 PMC5025200

[4] Boscan-Duque LA, Arzalluz-Fisher AM, Ugarte C, Sanchez D, Wittum TE, Hoet AE. Reduced susceptibility to quinolones among Salmonella serotypes isolated from poultry at slaughter in Venezuela. J Food Prot. 2007;70(9): 2030–5. 17900079 10.4315/0362-028x-70.9.2030

[5] Castellanos LR, van der Graaf-van Bloois L, Donado-Godoy P, Leon M, Clavijo V, Arevalo A, et al Genomic Characterization of Extended-Spectrum Cephalosporin-Resistant Salmonella enterica in the Colombian Poultry Chain. Front Microbiol. 2018;9: 2431 10.3389/fmicb.2018.02431 30459720 PMC6232905

[6] Cejas D, Vignoli R, Quinteros M, Marino R, Callejo R, Betancor L, et al First detection of CMY-2 plasmid mediated beta-lactamase in Salmonella Heidelberg in South America. Rev Argent Microbiol. 2014;46(1): 30–3. 10.1016/S0325-7541(14)70044-6 24721271

[7] Donado-Godoy P, Bernal JF, Rodriguez F, Gomez Y, Agarwala R, Landsman D, et al Genome Sequences of Multidrug-Resistant Salmonella enterica Serovar Paratyphi B (dT+) and Heidelberg Strains from the Colombian Poultry Chain. Genome Announce. 2015;3(5). 10.1128/genomeA.01265-15 PMC4616196 26494672

[8] Fernandes SA, Camargo CH, Francisco GR, Bueno MFC, Garcia DO, Doi Y, et al Prevalence of Extended-Spectrum beta-Lactamases CTX-M-8 and CTX-M-2-Producing Salmonella Serotypes from Clinical and Nonhuman Isolates in Brazil. Microb Drug Resist. 2017;23(5): 580–9. 10.1089/mdr.2016.0085 27828759

[9] Fitch FM, Carmo-Rodrigues MS, Oliveira VG, Gaspari MV, Dos Santos A, de Freitas JB, et al beta-Lactam Resistance Genes: Characterization, Epidemiology, and First Detection of blaCTX-M-1 and blaCTX-M-14 in Salmonella spp. Isolated from Poultry in Brazil-Brazil Ministry of Agriculture's Pathogen Reduction Program. Microb Drug Resist. 2016;22(2): 164–71. 10.1089/mdr.2015.0143 26380894

[10] Giuriatti J, Stefani LM, Brisola MC, Crecencio RB, Bitner DS, Faria GA. Salmonella Heidelberg: Genetic profile of its antimicrobial resistance related to extended spectrum beta-lactamases (ESBLs). Microb Pathog. 2017;109: 195–9. 10.1016/j.micpath.2017.05.040 28578094

[11] Medeiros MA, Oliveira DC, Rodrigues Ddos P, Freitas DR. Prevalence and antimicrobial resistance of Salmonella in chicken carcasses at retail in 15 Brazilian cities. Rev Panam Salud Publica. 2011;30(6): 555–60. 10.1590/s1020-49892011001200010 22358402

[12] Tiba-Casas MR, Camargo CH, Soares FB, Doi Y, Fernandes SA. Emergence of CMY-2-Producing Salmonella Heidelberg Associated with IncI1 Plasmids Isolated from Poultry in Brazil. Microb Drug Resist. 2019;25(2): 271–6. 10.1089/mdr.2018.0044 30256175

[13] Voss-Rech D, Vaz CS, Alves L, Coldebella A, Leao JA, Rodrigues DP, et al A temporal study of Salmonella enterica serotypes from broiler farms in Brazil. Poult Sci. 2015;94(3): 433–41. 10.3382/ps/peu081 25595481

[14] European Food Safety Authority ECfDPaC. The European Union summary report on antimicrobial resistance in zoonotic and indicator bacteria from humans, animals and food in 2014. EFSA Journal. 2016;14(2): 4380. 10.2903/j.efsa.2018.5182 PMC7009656 32625816

[15] Liakopoulos A, Geurts Y, Dierikx CM, Brouwer MS, Kant A, Wit B, et al Extended-Spectrum Cephalosporin-Resistant Salmonella enterica serovar Heidelberg Strains, the Netherlands(1). Emerg Infect Dis. 2016;22(7): 1257–61. 10.3201/eid2207.151377 27314180 PMC4918182

[16] Foley SL, Nayak R, Hanning IB, Johnson TJ, Han J, Ricke SC. Population dynamics of Salmonella enterica serotypes in commercial egg and poultry production. Appl Environ Microbiol. 2011;77(13): 4273–9. 10.1128/AEM.00598-11 21571882 PMC3127710

[17] Clothier KA, Byrne BA. Phenotypic and Genotypic Characterization of Animal-Source Salmonella Heidelberg Isolates. J Vet Med. 2016;2016: 6380890 10.1155/2016/6380890 26881274 PMC4735902

[18] Brandwagt D, van den Wijngaard C, Tulen AD, Mulder AC, Hofhuis A, Jacobs R, et al Outbreak of Salmonella Bovismorbificans associated with the consumption of uncooked ham products, the Netherlands, 2016 to 2017. Euro Surveill. 2018;23(1). 10.2807/1560-7917.ES.2018.23.1.17-00335 PMC5765776 29317018

[19] Mair-Jenkins J, Borges-Stewart R, Harbour C, Cox-Rogers J, Dallman T, Ashton P, et al Investigation using whole genome sequencing of a prolonged restaurant outbreak of Salmonella Typhimurium linked to the building drainage system, England, February 2015 to March 2016. Euro Surveill. 2017;22(49). 10.2807/1560-7917.ES.2017.22.49.17-00037 PMC5727591 29233257

[20] Simon S, Trost E, Bender J, Fuchs S, Malorny B, Rabsch W, et al Evaluation of WGS based approaches for investigating a food-borne outbreak caused by Salmonella enterica serovar Derby in Germany. Food Microbiol. 2018;71: 46–54. 10.1016/j.fm.2017.08.017 29366468

[21] McDermott PF, Tyson GH, Kabera C, Chen Y, Li C, Folster JP, et al Whole-Genome Sequencing for Detecting Antimicrobial Resistance in Nontyphoidal Salmonella. Antimicrob Agents Chemother. 2016;60(9): 5515–20. 10.1128/AAC.01030-16 27381390 PMC4997858

[22] Mevius DJ, Dierikx CM, Veldman KT, van Essen-Zandbergen A, Kant A. Monitoring of Antimicrobial Resistance and Antibiotic Usage in Animal in the Netherlands in 2013. MARAN 2014.

[23] Bekal S, Berry C, Reimer AR, Van Domselaar G, Beaudry G, Fournier E, et al Usefulness of High-Quality Core Genome Single-Nucleotide Variant Analysis for Subtyping the Highly Clonal and the Most Prevalent Salmonella enterica Serovar Heidelberg Clone in the Context of Outbreak Investigations. J Clinical Microbiol. 2016;54(2): 289–95. 26582830 10.1128/JCM.02200-15 PMC4733192

[24] Zankari E, Allesoe R, Joensen KG, Cavaco LM, Lund O, Aarestrup FM. PointFinder: a novel web tool for WGS-based detection of antimicrobial resistance associated with chromosomal point mutations in bacterial pathogens. Journal Antimicrob Chemother. 2017;72(10): 2764–8. 29091202 10.1093/jac/dkx217 PMC5890747

[25] Stamatakis A. RAxML version 8: a tool for phylogenetic analysis and post-analysis of large phylogenies. Bioinformatics. 2014;30(9): 1312–3. 10.1093/bioinformatics/btu033 24451623 PMC3998144

[26] Pattengale ND, Alipour M, Bininda-Emonds OR, Moret BM, Stamatakis A. How many bootstrap replicates are necessary? J Comput Biol. 2010;17(3): 337–54. 10.1089/cmb.2009.0179 20377449

[27] Ferrari R, Galiana A, Cremades R, Rodriguez JC, Magnani M, Tognim MC, et al Plasmid-mediated quinolone resistance (PMQR) and mutations in the topoisomerase genes of Salmonella enterica strains from Brazil. Braz J Microbiol. 2013;44(2): 651–6. 10.1590/S1517-83822013000200046 24294265 PMC3833171

[28] Saltykova A, Wuyts V, Mattheus W, Bertrand S, Roosens NHC, Marchal K, et al Comparison of SNP-based subtyping workflows for bacterial isolates using WGS data, applied to Salmonella enterica serotype Typhimurium and serotype 1,4,[5],12:i. PloS one. 2018;13(2): e0192504. 29408896 10.1371/journal.pone.0192504 PMC5800660

[29] Edirmanasinghe R, Finley R, Parmley EJ, Avery BP, Carson C, Bekal S, et al A Whole-Genome Sequencing Approach To Study Cefoxitin-Resistant Salmonella enterica Serovar Heidelberg Isolates from Various Sources. Antimicrob Agents Chemother. 2017; 61(4). 10.1128/AAC.01919-16 PMC5365727 28137797

[30] Kuang D, Zhang J, Xu X, Shi W, Chen S, Yang X, et al Emerging high-level ciprofloxacin resistance and molecular basis of resistance in Salmonella enterica from humans, food and animals. Int J Food Microbiol. 2018;280: 1–9. 10.1016/j.ijfoodmicro.2018.05.001 29747038

[31] Song Q, Xu Z, Gao H, Zhang D. Overview of the development of quinolone resistance in Salmonella species in China, 2005–2016. Infect Drug Resist. 2018;11: 267–74. 10.2147/IDR.S157460 29520157 PMC5833789

[32] Zhang WH, Zhang CZ, Liu ZJ, Gu XX, Li W, Yang L, et al In Vitro Development of Ciprofloxacin Resistance of Salmonella enterica Serovars Typhimurium, Enteritidis, and Indiana Isolates from Food Animals. Microb Drug Resist. 2017;23(6): 687–94. 10.1089/mdr.2016.0119 28085562

[33] European Food Safety Authority ECfDPaC. The European Union summary report on antimicrobial resistance in zoonotic and indicator bacteria from humans, animals and food in 2016. EFSA Journal 2018;16(2): 5182. 10.2903/j.efsa.2018.5182 PMC7009656 32625816

[34] Laurel M. The National Antimicrobial Resistance Monitoring System: NARMS Integrated Report, 2015. US Department of Health and Human Services, FDA 2017.

[35] Noda T, Murakami K, Etoh Y, Okamoto F, Yatsuyanagi J, Sera N, et al Increase in resistance to extended-spectrum cephalosporins in Salmonella isolated from retail chicken products in Japan. PloS one. 2015;10(2): e0116927 10.1371/journal.pone.0116927 25642944 PMC4314076

[36] Botelho LA, Kraychete GB, Costa e Silva JL, Regis DV, Picao RC, Moreira BM, et al Widespread distribution of CTX-M and plasmid-mediated AmpC beta-lactamases in Escherichia coli from Brazilian chicken meat. Mem Inst Oswaldo Cruz. 2015;110(2): 249–54. 10.1590/0074-02760140389 25946250 PMC4489457

[37] Shaikh S, Fatima J, Shakil S, Rizvi SM, Kamal MA. Antibiotic resistance and extended spectrum beta-lactamases: Types, epidemiology and treatment. Saudi J Biol Sci. 2015;22(1): 90–101. 10.1016/j.sjbs.2014.08.002 25561890 PMC4281622

[38] Bonelli RR, Moreira BM, Picao RC. Antimicrobial resistance among Enterobacteriaceae in South America: history, current dissemination status and associated socioeconomic factors. Drug Resist Updat. 2014;17(1–2): 24–36. 10.1016/j.drup.2014.02.001 24618111

[39] Sampaio JL, Gales AC. Antimicrobial resistance in Enterobacteriaceae in Brazil: focus on beta-lactams and polymyxins. Braz J Microbiol. 2016;47 Suppl 1: 31–7. 27825605 10.1016/j.bjm.2016.10.002 PMC5156504

[40] Moura Q, Fernandes MR, Silva KC, Monte DF, Esposito F, Dropa M, et al Virulent nontyphoidal Salmonella producing CTX-M and CMY-2 beta-lactamases from livestock, food and human infection, Brazil. Brazil. Virulence. 2018;9(1): 281–6. 10.1080/21505594.2017.1279779 28102761 PMC5955470

[41] Mendonca EP, de Melo RT, Nalevaiko PC, Monteiro GP, Fonseca BB, Galvao NN, et al Spread of the serotypes and antimicrobial resistance in strains of Salmonella spp. isolated from broiler. Braz J Microbiol. 2019;50(2): 515–22. 10.1007/s42770-019-00054-w 31001793 PMC6863245

[42] Gouvêa R, Santos F, dos Santos Machado L, Henrique Nunes Panzenhagen P, Aquino M, Rosendo do Nascimento E, et al Fluoroquinolones in Industrial Poultry Production, Bacterial Resistance and Food Residues: a Review. Braz J Poultry Sci. 2015;17(1): 1–10.

[43] Roth N, Kasbohrer A, Mayrhofer S, Zitz U, Hofacre C, Domig KJ. The application of antibiotics in broiler production and the resulting antibiotic resistance in Escherichia coli: A global overview. Poult Sci. 2019;98(4): 1791–804. 10.3382/ps/pey539 30544256 PMC6414035

[44] Roth N, Mayrhofer S, Gierus M, Weingut C, Schwarz C, Doupovec B, et al Effect of an organic acids based feed additive and enrofloxacin on the prevalence of antibiotic-resistant E. coli in cecum of broilers. Poult Sci. 2017;96(11): 4053–60. 10.3382/ps/pex232 29050428

[45] Francino MP. Antibiotics and the Human Gut Microbiome: Dysbioses and Accumulation of Resistances. Front Microbiol. 2015;6: 1543 10.3389/fmicb.2015.01543 26793178 PMC4709861

[46] Gast RK, Regmi P, Guraya R, Jones DR, Anderson KE, Karcher DM. Colonization of internal organs by Salmonella Enteritidis in experimentally infected laying hens of four commercial genetic lines in conventional cages and enriched colony housing. Poult Sci. 2019;98(4): 1785–90. 10.3382/ps/pey541 30535349

[47] Santin E, Hayashi RM, Wammes JC, Gonzalez-Esquerra R, Carazzolle MF, Freire CCM, et al Phenotypic and Genotypic Features of a Salmonella Heidelberg Strain Isolated in Broilers in Brazil and Their Possible Association to Antibiotics and Short-Chain Organic Acids Resistance and Susceptibility. Front Vet Sci. 2017;4: 184 10.3389/fvets.2017.00184 29164140 PMC5671994

[48] Dias MR, Cavicchioli VQ, Camargo AC, Lanna FG, Pinto PS, Bersot Ldos S, et al Molecular tracking of Salmonella spp. in chicken meat chain: from slaughterhouse reception to end cuts. J Food Sci Technol. 2016;53(2): 1084–91. 10.1007/s13197-015-2126-3 27162388 PMC4837711

[49] Yamatogi RS, Oliveira HC, Possebon FS, Pantoja JC, Joaquim JG, Pinto JP, et al Qualitative and Quantitative Determination and Resistance Patterns of Salmonella from Poultry Carcasses. J Food Prot. 2016;79(6): 950–5. 10.4315/0362-028X.JFP-15-489 27296598

[50] Hancock SJ, Phan MD. Identification of IncA/C Plasmid Replication and Maintenance Genes and Development of a Plasmid Multilocus Sequence Typing Scheme. Antimicrob Agents Chemother. 2017;61(2). 10.1128/AAC.01740-16 PMC5278728 27872077

[51] Lindsey RL, Fedorka-Cray PJ, Frye JG, Meinersmann RJ. Inc A/C plasmids are prevalent in multidrug-resistant Salmonella enterica isolates. Appl Environ Microbiol. 2009;75(7): 1908–15. 10.1128/AEM.02228-08 19181840 PMC2663206

[52] Carattoli A, Villa L, Poirel L, Bonnin RA, Nordmann P. Evolution of IncA/C blaCMY-(2)-carrying plasmids by acquisition of the blaNDM-(1) carbapenemase gene. Antimicrob Agents Chemother. 2012;56(2): 783–6. 10.1128/AAC.05116-11 22123704 PMC3264282

[53] Folster JP, Grass JE, Bicknese A, Taylor J, Friedman CR, Whichard JM. Characterization of Resistance Genes and Plasmids from Outbreaks and Illness Clusters Caused by Salmonella Resistant to Ceftriaxone in the United States, 2011–2012. Microb Drug Resist. 2017;23(2): 188–93. 10.1089/mdr.2016.0080 27828730 PMC5656985

[54] Zhang XZ, Lei CW, Zeng JX, Chen YP, Kang ZZ, Wang YL, et al An IncX1 plasmid isolated from Salmonella enterica subsp. enterica serovar Pullorum carrying blaTEM-1B, sul2, arsenic resistant operons. Plasmid. 2018;100: 14–21. 10.1016/j.plasmid.2018.09.007 30248363

[55] Petrovska L, Mather AE, AbuOun M, Branchu P, Harris SR, Connor T, et al Microevolution of Monophasic Salmonella Typhimurium during Epidemic, United Kingdom, 2005–2010. Emerg Infect Dis. 2016;22(4): 617–24. 10.3201/eid2204.150531 26982594 PMC4806966

[56] Hoelzer K, Moreno Switt AI, Wiedmann M. Animal contact as a source of human non-typhoidal salmonellosis. Vet Res. 2011;42: 34 10.1186/1297-9716-42-34 21324103 PMC3052180

[57] Mughini-Gras L, Enserink R, Friesema I, Heck M, van Duynhoven Y, van Pelt W. Risk factors for human salmonellosis originating from pigs, cattle, broiler chickens and egg laying hens: a combined case-control and source attribution analysis. PloS one. 2014;9(2): e87933 10.1371/journal.pone.0087933 24503703 PMC3913680

