# Supplementary figures and images for: Characterization and whole genome sequencing of closely related multidrug-resistant Salmonella enterica serovar Heidelberg isolates from imported poultry meat in the Netherlands
Source: PLoS One. 2019 Jul 22;14(7):e0219795. doi: 10.1371/journal.pone.0219795 (PMC6645675; doi:10.1371/journal.pone.0219795)

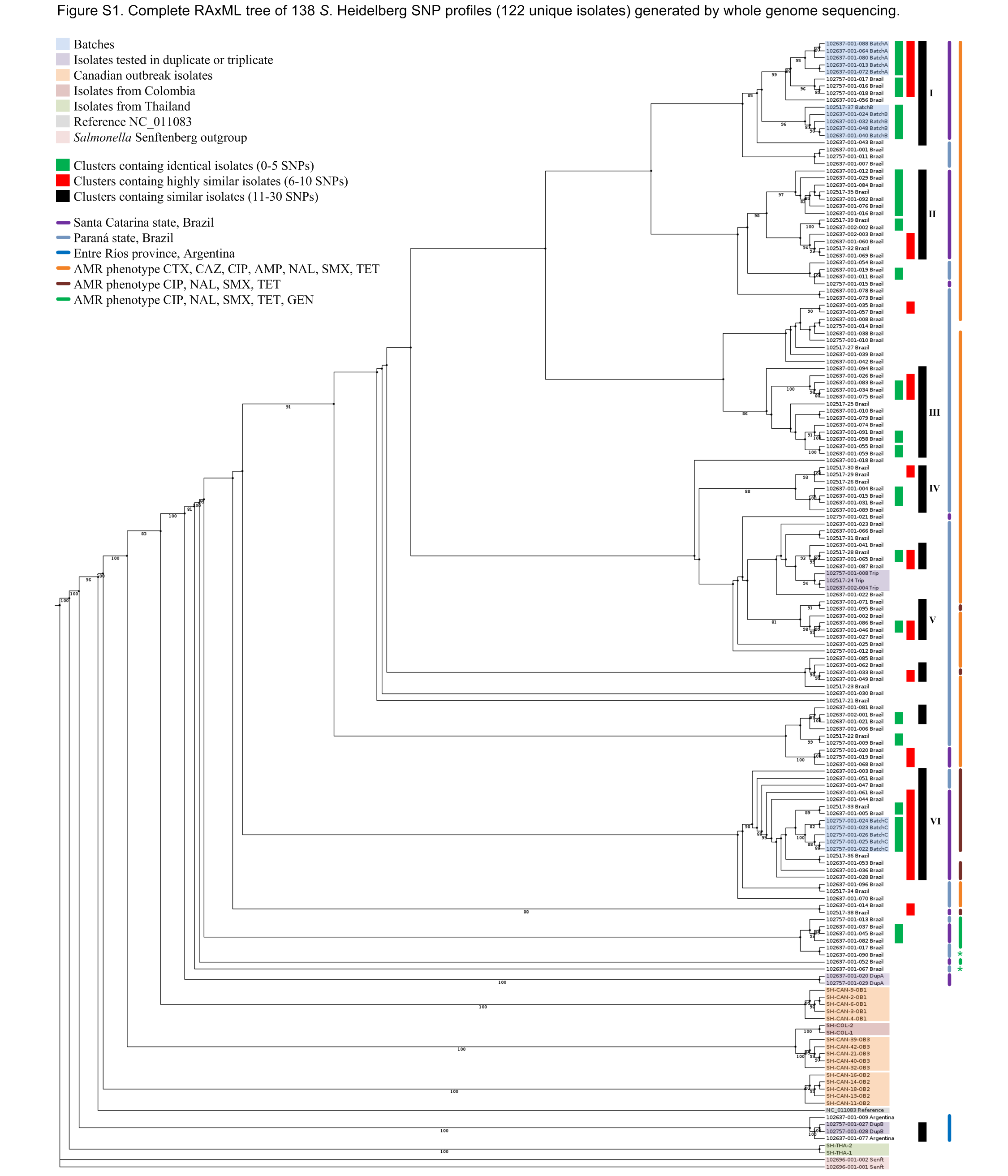

Supplement: S1 Fig — (TIF) [file pone.0219795.s001.tif]
